# Repellent and insecticidal efficacy of a new combination of fipronil and permethrin against the main vector of canine leishmaniosis in Europe (*Phlebotomus perniciosus*)

**DOI:** 10.1186/s13071-015-0683-y

**Published:** 2015-01-27

**Authors:** Pascal Dumont, Becky Fankhauser, Emilie Bouhsira, Emmanuel Lienard, Philippe Jacquiet, Frederic Beugnet, Michel Franc

**Affiliations:** Merial S.A.S., 29 Av Tony Garnier, 69007 Lyon, France; Institut National Polytechnique (INP) – Ecole Nationale Vétérinaire de Toulouse, 23 Chemin des Capelles, 31076 Toulouse, France

**Keywords:** Repellent, *Phlebotomus perniciosus*, Sandflies, Fipronil, Permethrin

## Abstract

**Background:**

Two successive laboratory experiments (A and B) were conducted to confirm the efficacy of a new fipronil and permethrin combination to repel and kill *Phlebotomus perniciosus* sandflies when applied once topically on dogs.

**Methods:**

Due to the difficulty to get enough available dogs and sandflies in one run, the study was divided into 2 experiments which had exactly the same design, and were conducted at the same place, with the same technicians. They compared dogs treated with a combination containing 67.6 mg/mL fipronil + 504.8 mg/mL permethrin (Frontect®/Frontline Tri-Act®, Merial) to untreated dogs. The treatments were applied topically once on Day 0. Sandfly exposures were performed on Days 1, 7, 14, 21 and 29 with 80 *P. perniciosus* female sandflies. After 60 min, sandflies were assessed for vitality and engorgement status. Live sandflies were kept in an insectary and observed for mortality counts 4 h after the exposure period ended.

**Results:**

Percent sandfly repellency on treated dogs was 98.2, 98.5, 99.2, 90.9 and 90.3%, for Days 1, 7, 14, 21, and 29, respectively. There was a significant difference (p ≤ 0.05) between the treated and control groups in both experiments and for the pooled data on every assessment day.

Insecticidal efficacy on treated dogs at 4 h post-exposure on Days 1, 7, 14, 21 and 29 was 98.7, 99.7, 96.8, 93.4, and 78.9%, respectively. There was a significant difference between the treated and control groups for live sandflies observed at 4 h post-exposure for all assessment days (p < 0.05).

**Conclusions:**

A single topical administration of a new combination of fipronil and permethrin demonstrated a significant repellent effect (i.e., > 80%) against *P. perniciosus* which lasted for 29 days after application. The repellent effect was accompanied by a significant insecticidal effect on sandflies. The results suggest that in endemic areas, the application of the fipronil-permethrin combination could be integrated into canine leishmaniosis prevention program.

## Abrégé

**Contexte:**

Deux études expérimentales successives (A et B) ont été conduites afin de confirmer l’efficacité d’une nouvelle combinaison de fipronil et de perméthrine pour repousser et tuer les phlébotomes *Phlebotomus perniciosus* après une application unique chez le chien.

**Méthodes:**

En raison de la difficulté à disposer de suffisamment de chiens et de phlébotomes en une fois, l’étude a été divisée en deux expérimentations ayant le même protocole, conduites au même endroit, et par les mêmes opérateurs. Elles ont comparées des chiens traités avec une combinaison contenant du fipronil à 67,6 mg/mL + de la perméthrine à 504,8 mg/mL (Frontect®/Frontline Tri-Act®, Merial) à des chiens non traités. Les traitements ont été appliqués en topique une fois au jour 0. Les expositions ont été réalisées aux jours 1, 7, 14, 21 et 29, avec 80 phlébotomes *P. perniciosus* femelles. Après 60 min, les phlébotomes ont été évalués pour leur vitalité et leur statut de gorgement. Les phlébotomes vivants ont été placés en insectarium et leur mortalité observée/comptabilisée 4 h après la fin de la période d’exposition.

**Résultats:**

Le pourcentage d’activité répulsive observé chez les chiens traités vis-à-vis des phlébotomes a été de 98,2, 98,5 99,2 90,9 et 90,3%, aux jours 1, 7, 14, 21 et 29, respectivement. A chaque jour d’évaluation, la différence était significative (p ≤0,05) entre les chiens traités et non traités, dans les deux expérimentations comme pour les données combinées.

Chez les chiens traités, l’efficacité insecticide 4 h post-exposition était de 98,7, 99,7, 96,8, 93,4, et 78,9% aux jours 1, 7, 14, 21 et 29. A chaque jour d’évaluation, la différence a été significative (p <0,05) entre les groupes traité et non traité quant au nombre de phlébotomes vivants 4 h post-exposition.

**Conclusions:**

Une seule administration topique de la nouvelle combinaison de fipronil et de perméthrine a démontré un effet répulsif significatif (i.e., > 80%) contre *P. perniciosus* durant 29 jours après l’application. L’effet répulsif était accompagné d’une action insecticide significative sur les phlébotomes. Les résultats suggèrent qu’en zone d’enzootie, l’application de la combinaison fipronil-perméthrine pourrait être intégrée dans un programme de prévention de la leishmaniose canine.

## Background

Leishmaniosis is a serious parasitic disease caused by flagellated protozoa of the genus *Leishmania*. The protozoa are transmitted to animals and humans by haematophagous female sandflies of the genus *Phlebotomus* in the Old World and *Lutzomyia* in the New World. Although certain wild mammals may be involved in the transmission of leishmaniosis, domestic dogs appear to be the principal reservoir of *Leishmania infantum* throughout the world [1]. In Europe, there is a tendency for leishmaniosis but also other canine vector-borne diseases to have an increased distribution [2]. This is related to several factors, including climate and social changes [3].

The prevention of canine leishmaniosis in dogs is based on several measures, including anti-*Leishmania* vaccines and methods for protecting healthy dogs against sandfly bites [4,5]. The studies reported here were conducted to assess the repellent and insecticidal efficacies of a new spot on topical combination of fipronil and permethrin (Frontect®/Frontline Tri-Act®, Merial) against the main vector of canine leishmaniosis in Europe (*Phlebotomus perniciosus*). Such a combination is intended to provide both repellent and insecticidal-acaricidal effects against several ectoparasites of dogs [6,7].

## Methods

Two successive experiments were conducted at the Ecole Nationale Vétérinaire de Toulouse. The 2 experiments were necessary due to the difficulty to include many dogs and to produce enough sandfly. Experiment A was also considered as a preliminary exploratory study whereas Experiment B was conducted according to Good Clinical Practices (GCP) as described in the International Cooperation on Harmonisation of Technical Requirements for Registration of Veterinary Medicinal Products (VICH) guideline 9 [8].

### Animals

Adult Beagle dogs were used in the experiements and had not been exposed to ectoparasiticides having a monthly efficacy or shorter 3 months prior to treatment, and were never exposed to long lasting ectoparasiticides (Experiment A (Exp. A): 6 males and 4 females, 10.7 to 11.4 months of age, weighing 6.9–9.2 kg; Experiment B (Exp. B): 8 males and 8 females, 14.1 to 14.9 months of age, weighing 8.1–10.4 kg). The dogs were housed individually in stainless steel cages with an exercise area, under controlled environmental conditions, fed with a commercial dry dog food ration, with water available *ad libitum*. No concurrent medication was given during the study. They were managed similarly and with due regard for their well-being. Animals were handled in compliance with Merial Animal Care and Use Guidelines and in compliance with the French regulatory requirements and ethical committee of Toulouse Veterinary School. The dogs were acclimated to the study conditions for at least 11 days prior to treatment and were observed for general health conditions throughout the study.

### Sandfly exposures

Sandfly exposures were performed one week prior to treatment for allocation purposes, and after treatment on Days 1, 7, 14, 21 and 29. Prior to each exposure, animals were anesthetized with intra-muscular injections of 0.02 to 0.03 mg/kg dexmedetomidine (Dextomidor®, Pfizer), 3.8 to 6.8 mg/kg ketamine (Imalgene® 1000, Merial) and 0.46 to 0.65 mg/kg of Diazepam (Valium®, Roche); placed individually into a sandfly proof exposure cage; and exposed to 80 *P. perniciosus* unfed female sandflies (strain originated from Lisbon, Portugal and maintained under laboratory conditions for 9 years). After approximately 60 min, live sandflies were removed from the exposure cage, counted and categorized as engorged (fed) or non-engorged (unfed). Dead sandflies remaining in the exposure cage or on the dog were counted and categorized as engorged or non-engorged. The dogs were then returned to their normal housing. Live sandflies recovered from each exposure (except pre-treatment) were maintained at appropriate environmental conditions for approximately 4 h after the exposure period ended, and dead phlebotomes were recovered and counted from each container.

### Allocation and treatment

To allocate the dogs, blocks were formed, based on descending pre-treatment counts of fed sandflies and 26 dogs were randomly allocated to the two treatment groups (10 for Exp. A and 16 for Exp. B). Dogs in Group 1 served as untreated control dogs. Dogs in Group 2 were treated on Day 0 with the topical combination of permethrin and fipronil. In Exp. A, 5 dogs were treated at the minimum recommended dose (0.1 mL/kg based on Day 0 body weight, corresponding to a dose of 6.73 mg/kg fipronil and 50.16 mg/kg permethrin) whereas in Exp. B, 8 dogs were treated at the recommended commercial dose (1.0 mL for dogs <10.0 kg, and 2.0 mL for dogs >10.0 to 20 kg, based on Day 0 body weight, delivering a minimum dose of 6.76 mg/kg fipronil and 50.48 mg/kg permethrin).

The dose was applied by parting the hair and applying the formulation directly onto the skin on the dorsal midline of the neck. The total volume was divided into two approximately equal portions. One fraction was applied between the base of the skull and the shoulder blades and the other fraction was applied at the front of the shoulder blades. The dogs were observed prior to treatment and hourly for 4 h following treatment administration.

### Data analysis

#### Percent sandfly repellency

The total number of engorged (alive + dead) sandflies at the end of each post-treatment exposure period was transformed to the natural logarithm of (count + 1) for calculation of geometric means (GM) by treatment group. Percent repellency was expressed as the percent reduction in fed sandflies of the treated group compared to the control group at each post-treatment exposure day: 100 × [(C-T)/C], where C is the GM of the control group and T is the GM of the treated group.

#### Percent insecticidal efficacy

All sandflies were collected at 60 min after exposure and classified as dead or alive. The live sandflies were put in containers and observed at 4 h post-exposure. The number of live sandflies after each post-treatment exposure was calculated by subtracting the number of dead sandflies at 4 h and the number of dead sandflies observed at the end of the 60 min exposure. The number of live sandflies was transformed to the natural logarithm of (count + 1) for calculation of geometric means. Percent insecticidal efficacy at 4 h post-exposure was calculated as 100 × [(C-T)/C], where C is the mean of live sandflies in the control group and T is the mean in the treated group.

The treated group was compared to the control group using Friedman’s rank test with blocks defined as the allocation blocks. The testing was two-sided and used a significance level of 5%.

## Results

No health abnormalities related to treatment were observed throughout the studies, including during hourly observations conducted for 4 h immediately after treatment.

In Exp. B, Dog 6277 in the control group vomited on Day 0 and then was normal through Day 7. The dog did not eat well from Days 8 to 14 and was not considered to be suitable for anesthesia and subsequent exposure to sandflies on Day 14. An intussusception was observed at ultrasonography on Day 15 and the dog was removed from the study, therefore the control group of Exp. B moved from 8 to 7 dogs on Days 14, 21 and 29.

Untreated control dogs had high numbers of engorged sandflies at the end of the exposure period at all time-points with means between 54.6 and 68.2 out of 80 (Table 1). With at least 68% of feeding behaviour on control dogs, it showed a robust sandfly strain population. The survival rate was also very good with at least 73.1% sandflies surviving until 4 h after the end of the exposure times in the control group (Table 2).

**Table 1:** **Percent repellency of** ***Phlebotomus perniciosus*** **in dogs treated with the combination of fipronil and permethrin (Experiments A & B) based on geometric means**

	**Number of engorged sandflies**					
**Exposure day experiment (A/B)**	**Dogs (n)**	**Untreated dogs**	**Dogs (n)**	**Treated dogs**	**Repellency (%)**	
1 (A)	5	60.0	5	3.4	94.4*	**98.2%** *
1 (B)	8	64.4	8	0.4	99.4*	
7 (A)	5	60.5	5	3.2	94.7*	**98.5%** *
7 (B)	8	61.3	8	0.2	99.7*	
14 (A)	5	59.3	5	0.4	99.3*	**99.2%** *
14 (B)	7	63.1	8	0.6	99.1*	
21 (A)	5	54.8	5	7.3	86.6*	**90.9%** *
21 (B)	7	64.7	8	4.6	92.9*	
29 (A)	5	54.6	5	1.5	97.3*	**90.3%** *
29 (B)	7	68.2	8	12.6	81.5*	

*Significant difference between the population means of the treated and control groups (p < 0.05).

Right column gives the % repellency based on geometric means obtained for each dog, pooling the two experiments, it is not the simple mean of the two % of repellency calculated for each experiment.

**Table 2:** **Percent insecticidal efficacy against** ***Phlebotomus perniciosus*** **observed at 4 h in dogs treated with the combination of fipronil and permethrin (Experiments A & B) based on Geometric means**

	**Number of live sandflies**					
**Exposure day experiment (A/B)**	**Dogs (n)**	**Untreated dogs**	**Dogs (n)**	**Treated dogs**	**Efficacy (%)**	
1 (A)	5	74.3	5	0.3	99.6*	**98.7%** *
1 (B)	8	73.5	8	1.6	97.9*	
7 (A)	5	75.2	5	0	100*	**99.7%** *
7 (B)	8	72.1	8	0.4	99.5*	
14 (A)	5	74.5	5	5.7	92.3*	**96.8%** *
14 (B)	7	72.1	8	1.2	98.4*	
21 (A)	5	75.2	5	33.9	54.9*	**93.4%** *
21 (B)	7	74.0	8	1.0	98.7*	
29 (A)	5	74.2	5	7.0	90.6*	**78.9%** *
29 (B)	7	75.1	8	25.7	65.7*	

*Significant difference between the population means of the treated and control groups (p < 0.05).

Right column gives the % insecticidal activity based on geometric means obtained for each dog, pooling the two experiments, it is not the simple mean of the two % of insecticidal activity calculated for each experiment.

### Repellency

Treated dogs had significantly fewer fed sandflies at the end of the exposure period than untreated control dogs for all study days (Exp. A, p = 0.025, Exp. B, p ≤ 0.008). The percent sandfly repellency after 60 min exposure was 98.2, 98.5, 99.2, 90.9 and 90.3% (Table 1), for Days 1, 7, 14, 21, and 29, respectively.

### Insecticidal efficacy

Treated dogs had significantly more dead sandflies at 4 h post-exposure than untreated control dogs for all challenge days (Exp. A, p = 0.025, Exp. B, p ≤ 0.008).

Percent insecticidal efficacy of the treated group at 4 h compared to the control group on Days 1, 7, 14, 21 and 29 was 98.7, 99.7, 96.8, 93.4, and 78.9% (Table 2), respectively. In Exp. A, the insecticidal efficacy was 54.9% on Day 21 and got back to 90.6% on Day 29.

## Discussion

The number of female sandflies used for each exposure was limited to 80 instead of a more classical number of 100 females [8,9] because of the large number of dogs in each study (10 dogs in Exp. A and 16 dogs in Exp. B) and the difficulty of rearing large numbers of female sandflies under laboratory conditions. There was no impact on the outcome of the studies due to the high rates of feeding and viability in the control groups.

The feeding behaviour of the sandflies on control dogs showed a robust population on all exposures days with at least 68% having fed after 60 min in the control group. Sandfly survival was also very good with at least 73.1% surviving until 4 h after the end of the exposure times in the control group.

The potential for a topical product to provide protection against sandfly-transmitted diseases depends on its ability to prevent the sandflies from taking a blood meal. Hence, the prevention of sandfly feeding was the major focus of this study. The new combination product showed a high repellency rate over one month with a plateau lasting until 21 days post-treatment (≥99.2%) and a progressive decrease to 90.3% on Day 29. A repellency over 80% is considered as a minimum threshold by the registration agencies and other authors [8,9]. This level of efficacy was also observed in other similar studies testing topical combinations including permethrin. Miro et al. [10] evaluated the activity of imidacloprid + permethrin (Advantix®) against *P. perniciosus* and reported ≥96.45% until Day 14, 92.73% on Day 21 and 74.7% on Day 28. Lienard et al. [11] assessed the repellent efficacy of another topical combination of dinotefuran + permethrin + pyriproxyfen (Vectra 3D), under the same experimental conditions, in the same location, and with the same *Phlebotomus* strain as in the present investigation. The repellency was 96.9, 99.7, 98.7, 83.5 and 87.0% on days 1, 7, 14, 21 and 28, respectively. The mortality effect was 97.8, 99.8, 73.7, 27.5 and 39.6% on days 1, 7, 14, 21 and 28, respectively. Against *P. papatasi*, Mencke et al. [12] found Advantix® efficacy to be ≥93.3% until Day 8, then 80.0, 72.8 and 55.9% on Days 15, 22 and 29, respectively. Molina et al. [13] tested a topical combination of permethrin alone (Exspot®) against *P. perniciosus* and reported efficacy ≥93.4% until Day 8 then 86.8, 67.6 and 61.0% on Days 15, 22 and 29, respectively. The observed results for the new topical combination of fipronil and permethrin are clearly close to what has been already published with other spot on formulation containing permethrin.

Insecticidal efficacy, as a secondary parameter, was high until Day 21 (93.4%), decreasing to 78.9% on Day 29. The insecticidal effect can be attributed to the action of both fipronil and permethrin, whereas the repellent activity of the product is likely due to the permethrin. Even if not a direct comparison, the mortality effect looks higher with the fipronil-permethrin combination than with the dinotefuran-permethrin-pyriproxyfen [7,11], under the same experimental conditions in the same laboratory and with the same sandfly strain.

In Exp. A, the insecticidal activity dropped on Day 21 to 54.9% and was 90.6% on Day 29, whereas it was 98.7% on Day 21 in Exp. B and then 65.7% on Day 29. The regular decrease was as expected in Exp. B. The lower insecticidal activity observed in treated dogs in Exp. A on Day 21 is difficult to explain as no particular variation was observed for the repellent effect, and no variation in the controls. It shows how it is important to include enough dogs in a study: 5 dogs is too limited and the 8 dogs included in Exp. B were important to compensate these observations. We could argue that the number of dogs is probably not sufficient and that a repetition of studies, using the same design, could confirm observations. This is certainly why the registration agencies request at least 2 efficacy studies (called dose confirmations) to confirm an indication [8,9].

## Conclusions

The new combination of fipronil and permethrin demonstrated a significant repellent effect against *P. perniciosus* bites as soon as it was applied on the dogs, and its repellent efficacy lasted 4 weeks. The results suggest that in endemic areas, the regular application of the new combination could be integrated in a canine leishmaniosis prevention program.
